# w*TSA-CRAFT*: an open-access web server for rapid analysis of thermal shift assay experiments

**DOI:** 10.1093/bioadv/vbad136

**Published:** 2023-09-29

**Authors:** Victor Reys, Julien Kowalewski, Muriel Gelin, Corinne Lionne

**Affiliations:** Centre de Biologie Structurale (CBS), CNRS UMR 5048, Université de Montpellier, INSERM U 1054, 34090 Montpellier, France; Centre de Biologie Structurale (CBS), CNRS UMR 5048, Université de Montpellier, INSERM U 1054, 34090 Montpellier, France; Centre de Biologie Structurale (CBS), CNRS UMR 5048, Université de Montpellier, INSERM U 1054, 34090 Montpellier, France; Centre de Biologie Structurale (CBS), CNRS UMR 5048, Université de Montpellier, INSERM U 1054, 34090 Montpellier, France

## Abstract

**Motivation:**

The automated data processing provided by the *TSA-CRAFT* tool enables now to reach high throughput speed analysis of thermal shift assays. While the software is powerful and freely available, it still requires installation process and command line efforts that could be discouraging.

**Results:**

To simplify the procedure, we decided to make it available and easy to use by implementing it with a graphical interface via a web server, enabling a cross-platform usage from any web browsers. We developed a web server embedded version of the *TSA-CRAFT* tool, enabling a user-friendly graphical interface for formatting and submission of the input file and visualization of the selected thermal denaturation profiles. We describe a typical case study of buffer condition optimization of the biologically relevant APH(3')-IIb bacterial protein in a 96 deep-well thermal shift analysis screening.

**Availability and implementation:**

w*TSA-CRAFT* is freely accessible for noncommercial usage at https://bioserv.cbs.cnrs.fr/TSA_CRAFT.

## 1 Introduction

Characterizing protein stability is often central in a wide variety of biochemistry applications, including buffer optimization, protein crystallization, ligand screening and fragment-based drug design for drug discovery process (Vedadi *et al.* 2006, Reinhard *et al.* 2013). Among the various methods available, the thermal shift assay (TSA) allows to analyze protein melting temperature, Tm, by differential scanning fluorimetry, DSF (Boivin *et al.* 2013). It allows to measure the thermal stability of a protein under different conditions (buffers, salts, additives). It is also used for the screening of drugs based on the characteristic that proteins are often stabilized by the binding of ligands. An example of a typical TSA experiment consists of incubating samples for 10 min and then using a qRT-PCR machine to increase the temperature of the samples by 1°C per minute from 20 to 95°C. The denaturation of the protein is followed by the presence of a fluorescent dye (classically SYPRO^®^ Orange) exhibiting high affinity for hydrophobic parts of the protein that become available upon denaturation. Protein denaturation can also be measured using the intrinsic fluorescence of tryptophan and tyrosine residues in the protein. Detected at both 350 and 330 nm, these changes in fluorescence signal indicate modifications in the environment of the fluorescent amino acids associated with transitions in the folding state of the protein.

The increasing usage of TSA is due to its potential high-throughput capabilities, but analyzing qRT-PCR output data requires substantial efforts and curve fitting knowledge. To compensate this issue, different tools have been developed. eSPC (Burastero *et al.* 2021) is a recent online platform which contains several tools: MoltenProt (Kotov *et al.* 2021) for Tm determination and FoldAffinity for the determination of dissociation constant from DSF. MoltenProt also allows the determination of the slope of the thermal unfolding curve, which is equally crucial for protein stability (Kotov *et al.* 2021). Another tool, CalFitter 2.0, implemented on a web server, incorporates an algorithm that allows access to results without prior knowledge of the underlying mathematics (Kunka *et al.* 2022). Finally, *TSA-CRAFT*, for TSA—Curve Rapid and Automatic Fitting Tool (Lee *et al.* 2019), was developed for a very basic analysis of DSF data. This TSA-beginner-friendly tool is used for automated data processing and Boltzmann equation fitting of qRT-PCR result files, enabling a quicker analysis of standard TSA experiments out of 96 (or 384) wells plates and a colour-code output of the fitting quality. Although the tool is open source, cross platform, freely accessible and based on few shell and Perl scripts, the library dependencies, its installation and usage can still be discouraging, especially for users unfamiliar with terminal command lines.

For this reason, we decided to spare users from the installation processes and made a graphical interface for input file submission by embedding the software under a dedicated web server hosted in our laboratory. In addition, we developed a new tool to format the raw qRT-PCR output data into a valid input file for *TSA-CRAFT*, saving even more time and limiting the risk of errors. In this application note, we describe a free and accessible web server of the embedded *TSA-CRAFT* tool plus the formatting tool under the w*TSA-CRAFT* name, available at https://bioserv.cbs.cnrs.fr/TSA_CRAFT.

## 2 Methods

### 2.1 Format specification

Input files needed to run w*TSA-CRAFT* are similar to the one(s) required by *TSA-CRAFT* (highlighted in green in Supplementary Fig. S1; examples given in Supplementary files). It consists of a mandatory thermal shift analysis table formatted as comma separated value (csv), where the first column holds the temperatures at which data points were measured and all subsequent columns contain fluorescence measurements for each well.

An additional optional csv file can also be provided, enabling the annotation of the various wells, thus allowing to disambiguate their content while displayed. This file must contain only three columns (separated by commas); (i) the index for the wells (#Well_index), (ii) the human annotated condition (#Annotation), and (iii) the name for the well(s) serving as reference(s) (#Reference_well_index), separated by spaces if multiple of them are present in the experiment.

Finally, a second optional input enabling the overlaying of multiple wells (up to a maximum of eight) on the same graph. Wells to be combined must be separated by commas and also present in the analysis.

In addition to the standard input section required to run *TSA-CRAFT*, we added a supplementary input section (highlighted in pink at bottom right of home page in Supplementary Fig. S1). It automatically converts raw thermal shift output data into a valid *TSA-CRAFT* input csv file, which allows to reach one step further in the speed of usage. This format converter is dedicated to the transformation of qRT-PCR Mx3005P (Stratagene) output data and takes as input two text files exported from the experiment results, the dissociation and amplification curve files.

### 2.2 Implementation

We implemented the *TSA-CRAFT* tool on a virtual machine hosted in our laboratory and running under Ubuntu 22.04 LTS using Apache2 (v.2.4.29) server and few Python3 (v.3.6.8) cgi scripts to validate input files, launch the *TSA-CRAFT* Perl (v.5.26.1) scripts and postprocess html rendering. This allows a simplified tool usage protocol in a cross-platform manner from major web browsers (tested on Firefox, Edge, Chrome and Safari). w*TSA-CRAFT* is freely accessible for noncommercial usage at https://bioserv.cbs.cnrs.fr/TSA_CRAFT.

#### 2.2.1 Graphical interface

Information about the original *TSA-CRAFT* tool (7) are available, with access to the publication, scripts repository and user manual (highlighted in blue in Supplementary Fig. S1). A simple yet effective web interface allows a graphical rendering for input files submission on the home page. Next to each input sections, test files are provided for download. Finally, as the standard *TSA-CRAFT* output are already provided in html language, a small postprocessing of the result page is performed: local paths are modified to URLs, a supplementary download links is added in the header and an additional input section for later graph overlaying generation is made accessible.

#### 2.2.2 Download of the results

As we did not want to block users on the web server results, we are providing a zip archive of all the generated results by *TSA-CRAFT*. This archive contains all previously generated results (html result page with relative paths and related graph overlays) and therefore can be used in a local environment. We strongly suggest users to download their results after their analyses as we are cleaning old queries results from our server on a weekly basis, for obvious saving of hard drive storage space.

#### 2.2.3 Graph overlay plots

*TSA-CRAFT* provides an extra script (plot_multi_well_curves.pl) generating comparative graphs overlay for a set of two to eight wells. To take advantage of all options allowed by the tool, we decided to provide this supplementary option at multiple locations. First, immediately in the home page input section (green in Supplementary Fig. S1, as described above), in the *TSA-CRAFT* result page and also below the graph following a previous overlay. In the result page (Supplementary Fig. S2), we added a new input form, from which users are able to use again this function to generate unlimited multi-well graphs, without computing again the overall results. In addition, well coordinates can be entered more easily by clicking directly on the ‘+’ sign next to the well identifier.

Links to download the various graphs produced are stacked at the bottom of the result page (Supplementary Fig. S2). Upon graph generation, the content of the zip archive is also updated with the newly made one.

Finally, we observed that wells annotated with more than 20 characters were going out of the graph. To compensate this issue, a small computation is performed, proportionally lowering the size of characters (up to a minimum of size 4) in the legend of the figures, hence allowing longer description of annotated wells.

#### 2.2.4 Raw data format converting tool

We are providing an additional format converting tool, enabling the automated generation of a valid *TSA-CRAFT* input csv file from raw qRT-PCR output data. To do so, first the dissociation file is parsed and cycles names are mapped to the corresponding temperatures. Then, the amplification file, which is holding fluorimetric information is parsed to gather amplitudes at each cycle. In order to be sure that temperatures correspond to the cycles, raw data should be exported without smoothing. Finally, we write a new csv file containing temperature values in the first column, followed by fluorescence amplitudes obtained for each well present in the experiment. We return the corresponding file as a downloadable csv file to the user, who simply needs to place it in the *TSA-CRAFT* input section. Thus, this new tool allows a faster and more secure conversion of raw data into an input file for *TSA-CRAFT*.

## 3 Results

**Figure 1.**
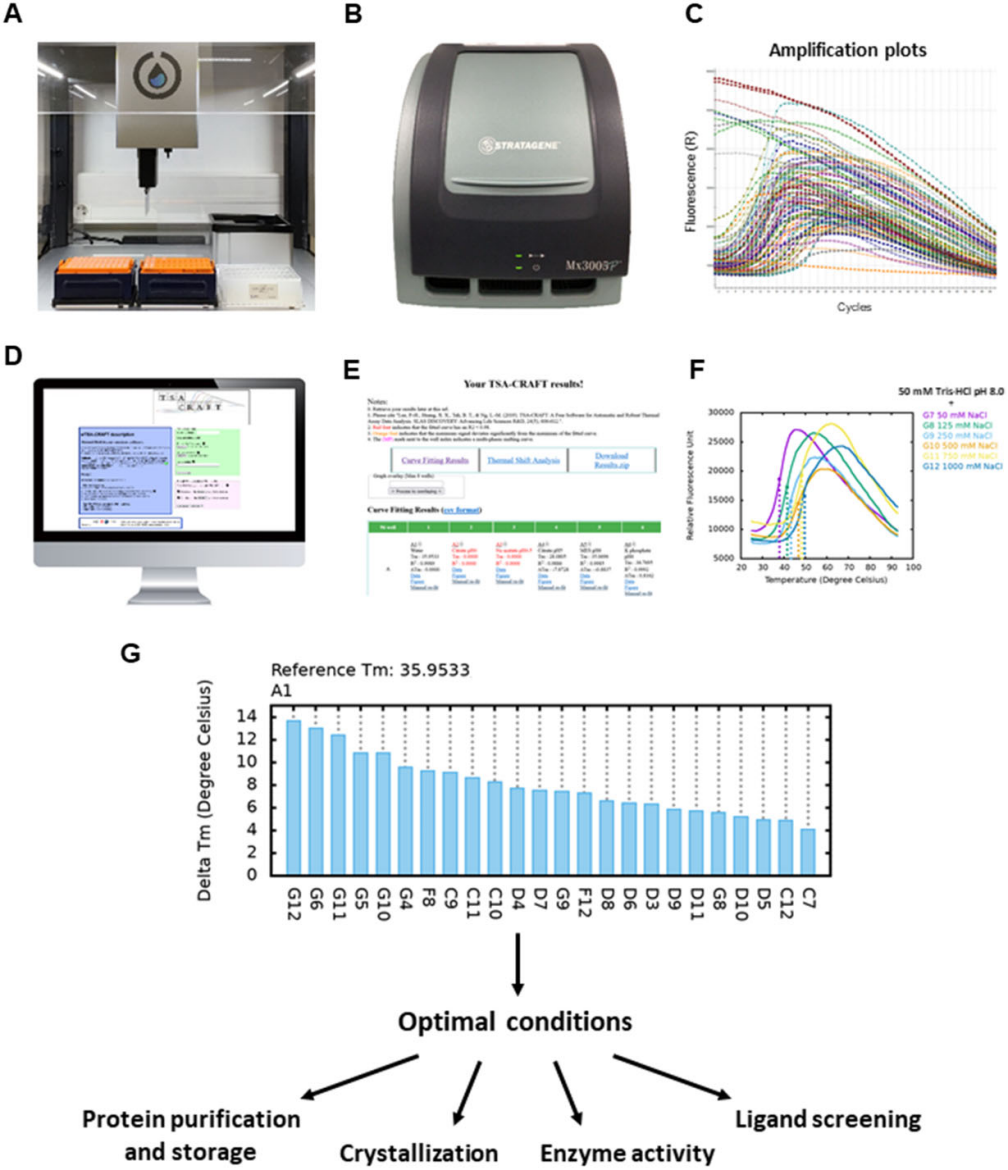
Workflow for the optimization of buffer conditions by TSA screening. (A) In a 96-well PCR plate, the protein is mixed with SYPRO^®^ Orange and different buffers using a pipetting robot. (B) The PCR plate is placed in a qRT-PCR device and warmed up to 95°C while the fluorescence is recorded. (C) The amplification and dissociation data are exported as text file in MxPro software. (D) The exported files are converted into a valid input csv file sent to w*TSA-CRAFT* (at https://bioserv.cbs.cnrs.fr/TSA_CRAFT). Curve fit analysis is performed by the embedded *TSA-CRAFT* software. (E) The results are used to compare the thermal stability of the protein in different buffer conditions. (F) Up to eight selected plots can be overlaid. (G) Optimal buffers are chosen accordingly to the requirements of the techniques used such as protein purification and storage, crystallization for X-ray structure determination, *in vitro* enzyme activity or ligand screening.

We applied a standard protocol for the screening by thermal shift assay of buffer conditions optimization for the protein APH(3')-IIb (UniProtKB_Id: Q9HWR2_PSEAE) of *Pseudomonas aeruginosa* implicated in bacterial resistance to aminoglycoside antibiotics (Wright 1999, Ramirez and Tolmasky 2010). We used the commercially available RUBIC Buffer Screen MD1-96 kit from Molecular Dimensions (3) which is presented as a 96 × 1 ml deep-well block. We have slightly adapted the protocol suggested by the manufacturer to fit the 20 μl tips of our Opentrons pipetting robot (Fig. 1A). We dispensed 4 μl of a mixture containing in house purified APH(3')-IIb (2.5 μM final) and SYPRO^®^ Orange (5× final), then added and mixed 20 μl of RUBIC Buffer Screen. The thermal denaturation screening was performed in a qRT-PCR device (Mx3005P, Stratagene) using a Cy3 and SYAL emission filter at a gain of 8 and 1 respectively (Fig. 1B). The fluorescence amplitudes obtained using the SYAL filter being saturated, those obtained with the Cy3 filter were used for further analysis.

Interestingly, we could rapidly identify the influence of NaCl concentration, allowing to reach higher melting temperatures (*T*_m_) with high concentrations (Fig. 1F, Supplementary Fig. S3A and D). Eventually, in well G12 (1 M NaCl, 0.05 M Tris–HCl at pH 8.0) the highest Tm of 49.6°C was reached, which corresponds to a Δ*T*_m_ of 13.7°C with respect to water (well A1).

Note that the graphs corresponding to pH values ≤ 4.5 (A2, A3, C2, C3, E1, and E2) could not be fitted with a sigmoid. The results are therefore shown in red, and inspection of the curves suggests that the protein is already denatured at 25°C. Curves at pH 5 could be fitted (A4, C4, and E4), but do not show a classic sigmoid thermal denaturation profile. Whichever tool is used to fit TSA curves, users should critically analyze the fits obtained so as not to over-interpret their data. In special cases, such as biphasic denaturation, other equations not available in *TSA-CRAFT* can be used, but require skill in interpreting the results (4–6). Alternatively, in the case of a multi-phasic melting curve, the user can determine each *T*_m_ manually by adjusting the fitting bounds for each sigmoid portion. For this, click on ‘Manual re-fit’ in the result page and choose the lower and upper temperature boundaries.

In our case study, pH lower than 5.5 were identified as deleterious for the protein thermal stability (Supplementary Fig. S4A and B).

## 4 Conclusion

These results allowed us to carry out several follow up works, by choosing the most suitable buffer for each technique taking into account the preferences of the studied protein (Fig. 1G). Thus, the protein was purified and stored in a buffer composed of 50 mM Tris–HCl pH 7.5, 40 mM KCl and 1 mM DTT. Its enzymatic activity was measured in the same buffer supplemented with 1 mM MgCl_2_. As Tris is not compatible with ITC affinity measurements, these were carried out in an equivalent buffer containing HEPES instead of Tris. Finally, the protein was crystallized with identified best ligands in 100 mM HEPES pH 7.4, 0.6 M tri-sodium citrate and 25 mM KCl. The choice of these buffers was guided principally by the TSA screening described above.

## Supplementary Material

vbad136_Supplementary_Data

## Data Availability

The data underlying this article are available in the article and in its online supplementary material.
