# Correction to: w*TSA-CRAFT*: an open-access web server for rapid analysis of thermal shift assay experiments

**DOI:** 10.1093/bioadv/vbae050

**Published:** 2024-04-09

This is a correction to: Victor Reys, Julien Kowalewski, Muriel Gelin, Corinne Lionne, w*TSA-CRAFT*: an open-access web server for rapid analysis of thermal shift assay experiments, *Bioinformatics Advances*, Volume 3, Issue 1, 2023, vbad136, https://doi.org/10.1093/bioadv/vbad136

In the originally published version, the grant from the Agence Nationale de la Recherche was incorrectly stated as ANR-17-CE18-0011. This has been corrected to read ANR-19-AMRB-0001-01.

