# Supplementary material for: wTSA-CRAFT: an open-access web server for rapid analysis of thermal shift assay experiments
Source: Bioinform Adv. 2023 Sep 29;3(1):vbad136. doi: 10.1093/bioadv/vbad136 (PMC10562953; doi:10.1093/bioadv/vbad136)
Supplement: vbad136_Supplementary_Data [file vbad136_supplementary_data.zip › Reys et al 2023 Supplementary material.docx]

Supplementary material

*Communication from the author of TSA-CRAFT authorizing its implementation:*

P. H. Lee (May 2020) TSA-CRAFT: Webserver Implementation. "Hi Victor, Thanks for that you are interested in and implementing our TSA-CRAFT. We have some private issue for maintenance of web version of TSA-CRAFT so that only the standalone version is released at the moment. I am glad that you are so kind and generous to spread TSA-CRAFT for us. Basically, I agree that you/your group to build a public TSA-CRAFT web server. Would you please clearly put the information on your homepage of TSA-CRAFT including the reference of TSA-CRAFT paper, the link of URL for standalone version in source forge and some statement like "the implementation is under agreement of the authors of TSA-CRAFT"? Regards, Po-Hsien Lee.".

*Detailed procedures for using wTSA-CRAFT can be found in the user manual:*

<https://bioserv.cbs.cnrs.fr/TSA_CRAFT/wTSA_CRAFT_manual.pdf>

*Examples of input files are given in the Supplementary files:*

- Input csv file: TSA_CRAFT_Input.csv

- Annotation file: Annotation.csv

- qRT-PCR Mx3005P output data: TSA PaeAP3 Rubic.mxp

- amplification curve file: Amplification.txt

- dissociation curve file: Dissociation.txt

*Supplementary figures:*


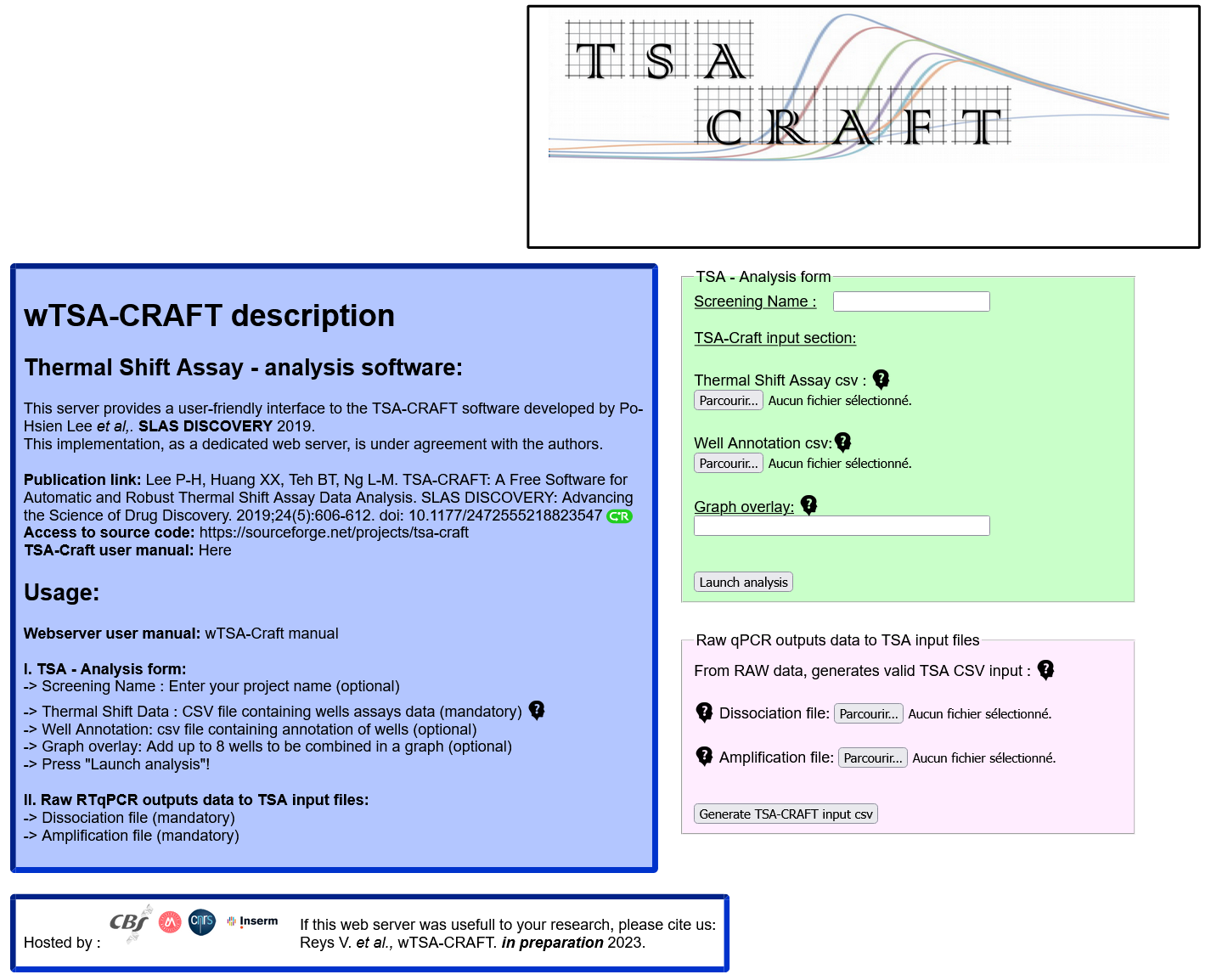


Figure S1. Screenshot of the w*TSA-CRAFT* home page at *https://bioserv.cbs.cnrs.fr/TSA_CRAFT/*. The different sections of the home page are highlighted by frames of different colours. On the left, in blue, information of the original *TSA-CRAFT* algorithm and access to the publication, software and user manual. On the top-right, in green, the TSA Analysis form, containing a text input to provide the name of the screening, enabling to properly name the later generated zip archive (optional), the mandatory TSA input file as csv, the well annotation csv (optional) and a text input for the combination of multiple graph overlay (optional). At the bottom-right, in pink, the form holding the two input file acceptor for the automated format conversion from raw qRT-PCR outputs from Mx3005P (Stratagene) to valid *TSA-CRAFT* input csv file by providing both the dissociation and amplification files.


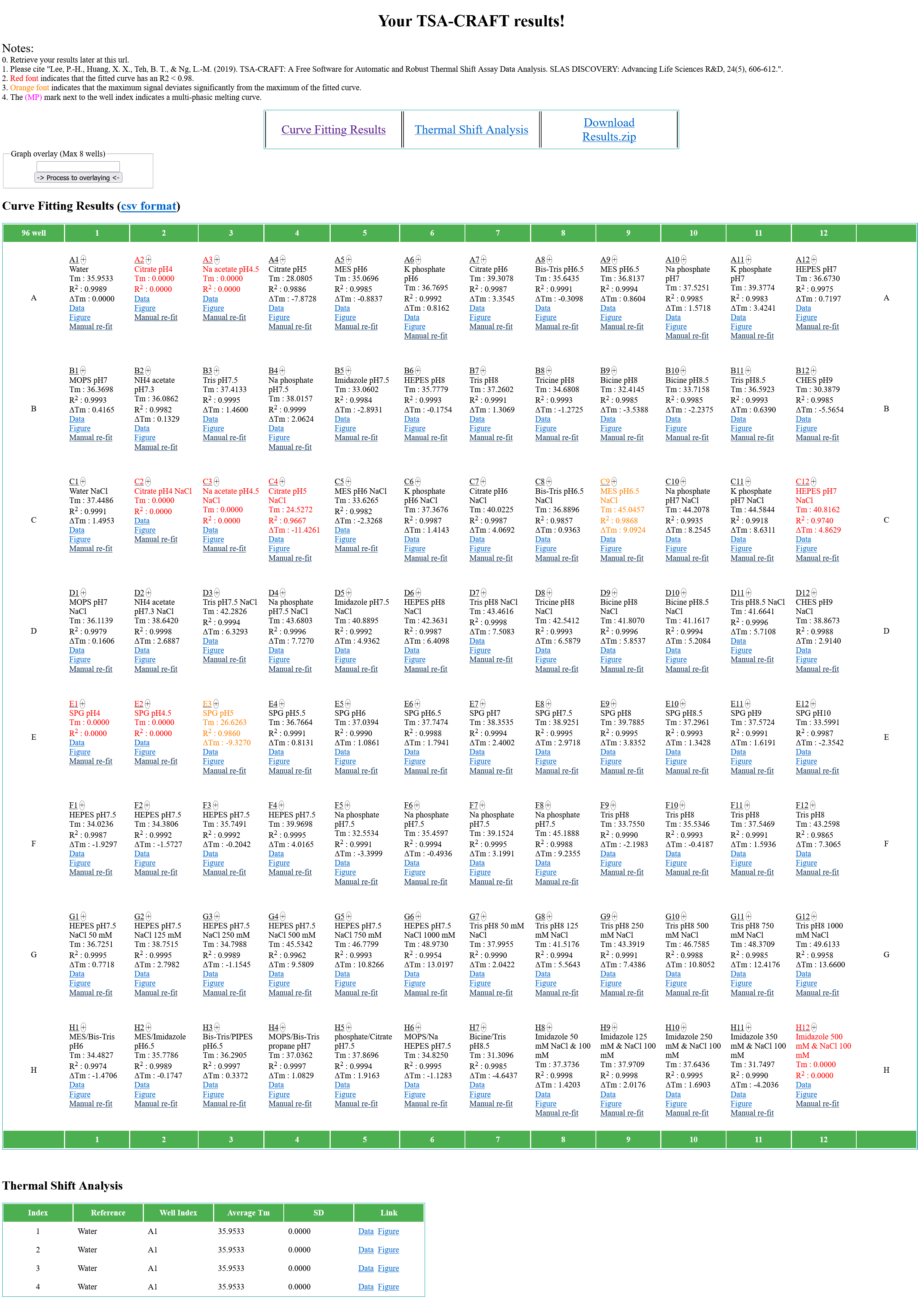


Figure S2. Screenshot of the edited *TSA-CRAFT* result page. In the header are available the various data files that can be downloaded. An additional link to a zip archive containing all results has been added by editing the original *TSA-CRAFT* output. A supplementary ‘Graph overlay’ combination input form is also provided, where the user can launch the ‘*plot_multi_well_curves.pl*’ script by providing up to 8 wells separated by comas or clicking on the ‘+’ sign next to the well identifiers. The result page is followed by a table holding the various thermal shift analysis for each well with a colour code related to the quality of the curve fit. Manual fitting is possible for each individual well with the ‘Manual re-fit’ option.

| **A**  **** | **B**  ****  **C**  **** |
| --- | --- |
| **D**   | **E**  **** |

Figure S3. Histograms of TSA T*_m_* data for all 96 wells. APH(3')-IIb thermal stability was measured in different conditions using the RUBIC Buffer kit (Molecular Dimensions): (A) buffers (100 mM) of different pHs at low (0 mM NaCl, blue bars) or high (250 mM NaCl, red bars) ionic strength, (B) composite buffer with extended range of pH deconvoluting pH from buffer effect, (C) buffers at different concentrations (20 to 250 mM), (D) different ionic strengths by variation of NaCl concentration (50 to 1000 mM) and (E) Combination of buffers and effect of imidazole (50 to 500 mM) in presence of 100 mM NaCl.
