# Supplementary material for: wTSA-CRAFT: an open-access web server for rapid analysis of thermal shift assay experiments
Source: Bioinform Adv. 2023 Sep 29;3(1):vbad136. doi: 10.1093/bioadv/vbad136 (PMC10562953; doi:10.1093/bioadv/vbad136)
Supplement: vbad136_Supplementary_Data [file vbad136_supplementary_data.zip › wTSA_CRAFT_user_manual.pdf]

wTSA-CRAFT User Manual

by Victor Reys, Julien Kowalewski, Muriel Gelin and Corinne Lionne
(Centre de Biologie Structurale, Montpellier)

September 2023

Atelier de
Biologie
Chimie
Informatique
Structurale

This user manual complements the TSA-CRAFT user manual by Lee et al., 2019, TSA-CRAFT user manual.
It includes guidance on control of fitting quality and present the new features.

The web server embedded version of the *TSA-CRAFT* tool, w*TSA-CRAFT*, enables a user-friendly graphical interface for formatting and submission of the input file and visualization of the selected thermal denaturation profiles. It can be found at: https://bioserv.cbs.cnrs.fr/TSA_CRAFT/

Starting with w*TSA-CRAFT*:

1- **Save the results in MxPro**

In the tab 'Analysis selection/setup', select all the wells to export, Replicates: Treat individually
In the tab 'Results', remove the smoothing: right click on the graphical area, Analysis term settings / Smoothing / uncheck the 2 boxes

Generate Amplification plots:

- Fluorescence R (Multicomponent view) or dR (with baseline correction)
- File / Export Chart Data / Export chart data to text file / Format 2 -- Horizontally grouped by plot
- Save the file amplification.txt

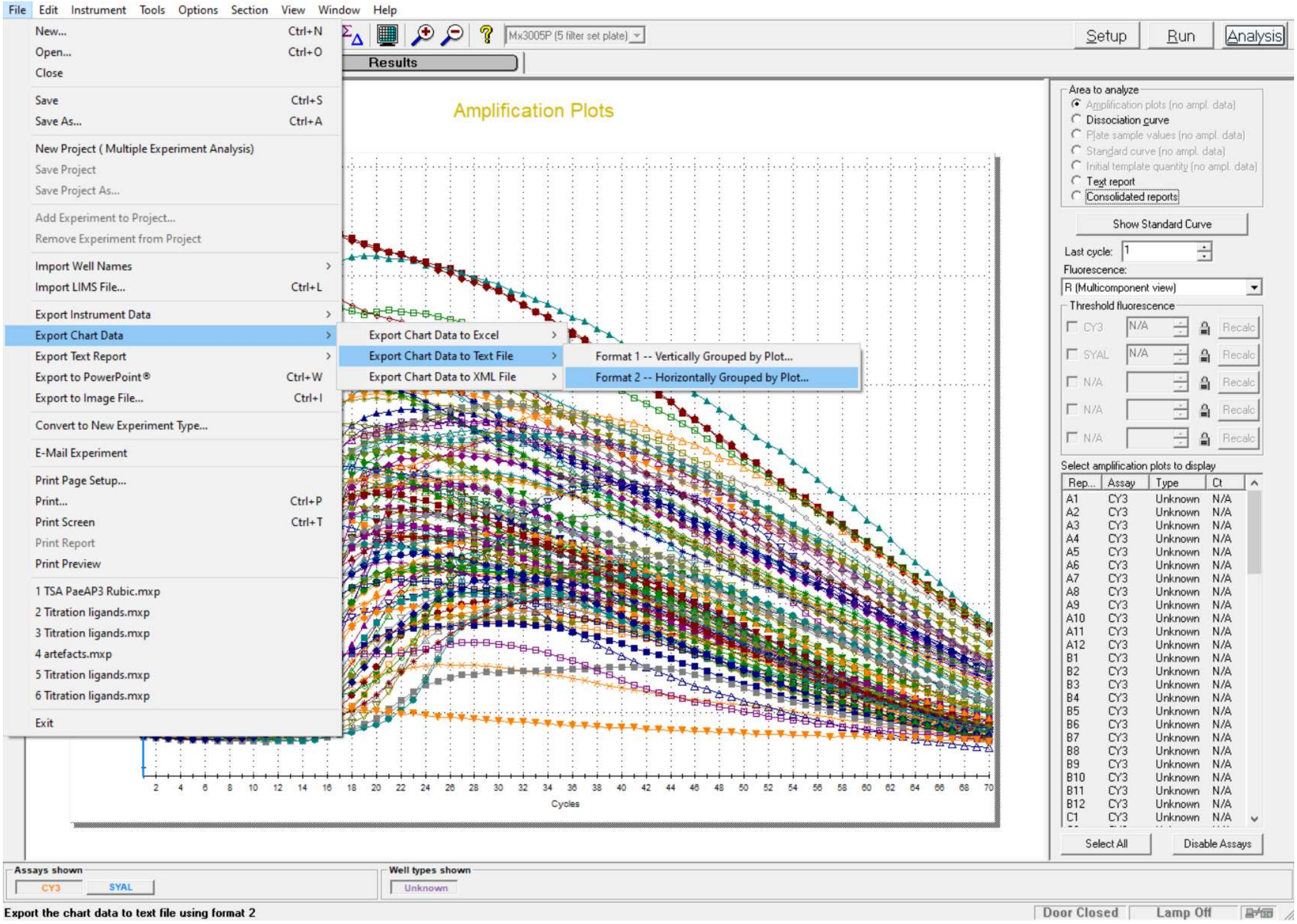

Generate Dissociation plots

This step allows to retrieve the temperature column instead of the cycle number

- Fluorescence R (Multicomponent view)
- File / Export Chart Data / Export chart data to text file / Format 2 -- Horizontally grouped by plot
- Save the file dissociation.txt

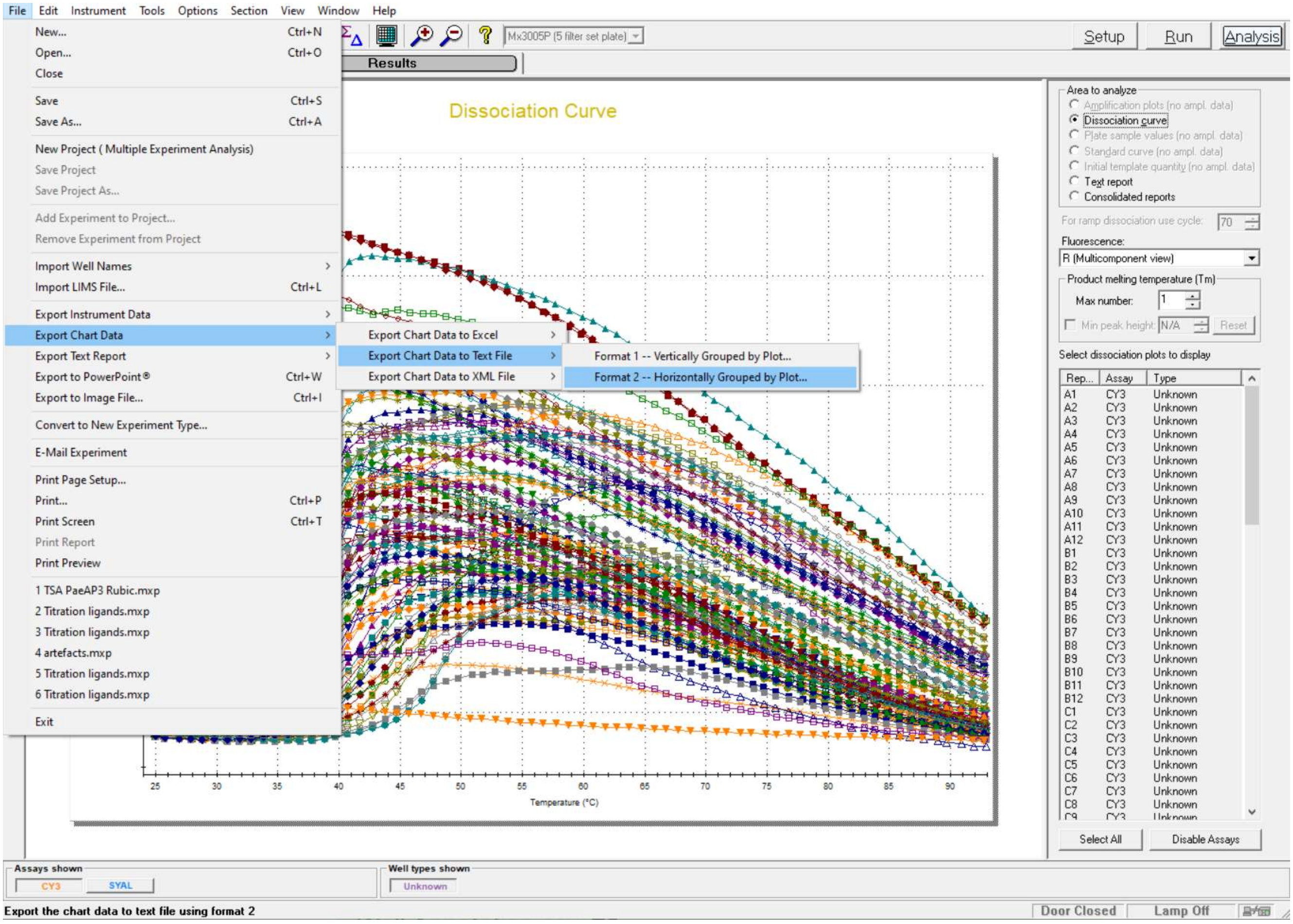

2- Convert raw RTqPCR outputs data to TSA input file

In the pink box of the raw data conversion module, upload the following files:

- Dissociation file (mandatory)
- Amplification file (mandatory)

This algorithm transforms raw data from RTqPCR MxPro3005P to a valid Thermal Shift Assay TSA-CRAFT input csv.

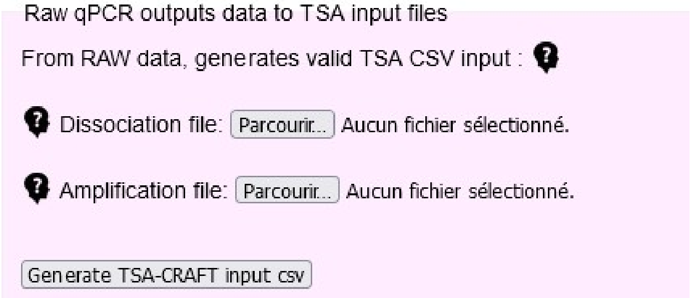

3- Launch the fittings using TSA-CRAFT

In the green box of the analysis form module, fill in the following information:

- Screening Name: Enter your project name (optional)
- Thermal Shift Data: CSV file containing wells assays data (mandatory). The file format is described in the TSA-CRAFT user manual and a template can be found here: Input CSV File Template
- Well Annotation: csv file containing annotation of wells (optional). The file format is described in the TSA-CRAFT user manual and a template can be found here: Annotation File Template
- Graph overlay: Add up to 8 wells to be combined in a graph (optional). Name of the wells to be combined, separated by commas (for example A1,A3,C3).

Then press "Launch analysis".

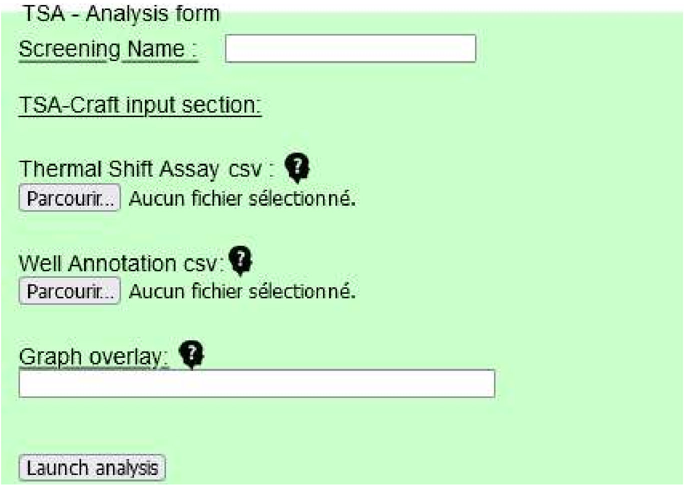

Please note that TSA-Craft CSV files must contain ',' (commas) to separate columns (';' is not allowed, but unfortunately is the default export type by Excel). Consider using LibreOffice or another software if you troubleshoot with this. Alternatively, replace semicolons with commas in a text editor.

4- Visualize the fitted curves

Each curve can be viewed individually by clicking on Figure on the results page.
It is also possible to represent several curves (maximum 8) on the same graph. This can be done by indicating the coordinates of the wells to be combined, spaced by a comma, at different stages: (1) before fitting under Graph overlay on the home page, (2) after fitting on the results page or (3) under the graph following a previous overlay.
On the results page, well coordinates can be entered more easily by clicking directly on the + sign next to the well identifier as shown below.

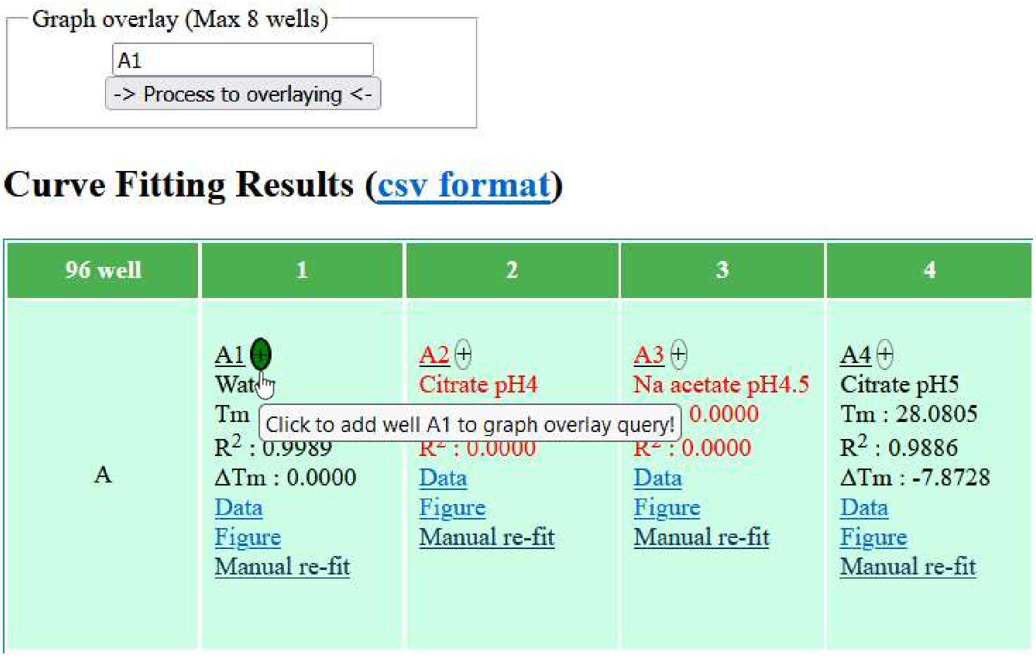

Curves can be saved as png files.

5- Interpret the results

As mentioned in the TSA-CRAFT user manual, denaturation parameters are fitted using the classical Boltzmann equation. Users should critically analyse the fits obtained so as not to over-interpret their data. In most cases, when fitting procedure fails with the classical Boltzmann equation, this means either that (1) the buffer is totally unsuitable for the protein (not properly folded at room temperature), (2) the ligand is incompatible with the method (e.g. DTT, detergent, ligands absorbing or fluorescing at wavelengths close the excitation of emission wavelengths of SYPRO® Orange), (3) the protein is not suitable for this technique (around 20% of proteins are not eligible). Alternatively, the problem may come from an experimental or pipetting mistakes. In these cases, it is preferable to use other methods or appropriate buffer conditions.
To help identify atypical curves, a colour code is given on the result page. Red font indicates that the fitted curve has an R^2 below 0.98. Orange font indicates that the maximum signal deviates significantly from the maximum of the fitted curve. Finally, the (MP) mark next to the well index indicates a multi-phasic melting curve, where the two discrete local maxima are found for R^2. In this case, the user can determine each Tm manually by adjusting the fitting bounds for each sigmoid portion). For this, click on Manual re-fit in the result page (see below) and choose the lower and upper temperature boundaries.

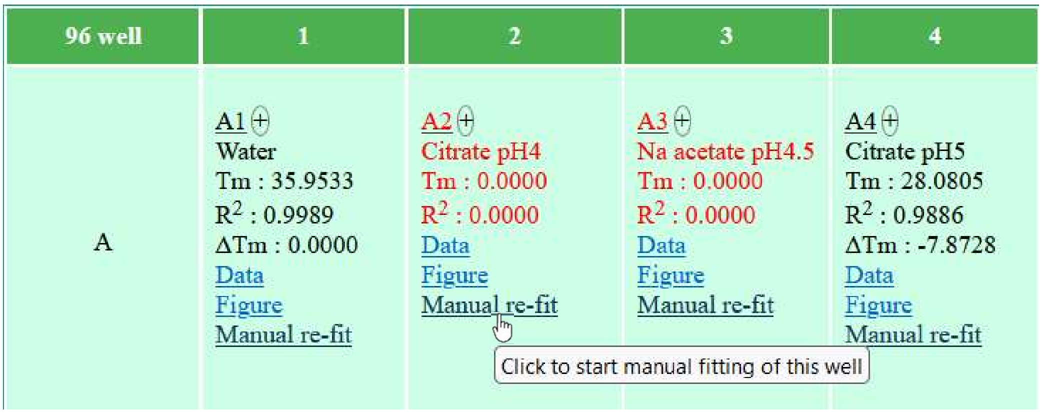

96 well	1	2	3	4
A	A1 ⊕<br>Water<br>Tm : 35.9533<br>R^2 : 0.9989<br>ΔTm : 0.0000<br>Data<br>Figure<br>Manual re-fit	A2 ⊕<br>Citrate pH4<br>Tm : 0.0000<br>R^2 : 0.0000<br>Data<br>Figure<br>Manual re-fit	A3 ⊕<br>Na acetate pH4.5<br>Tm : 0.0000<br>R^2 : 0.0000<br>Data<br>Figure<br>Manual re-fit	A4 ⊕<br>Citrate pH5<br>Tm : 28.0805<br>R^2 : 0.9886<br>ΔTm : -7.8728<br>Data<br>Figure<br>Manual re-fit

Click to start manual fitting of this well

6- Download of the results

Results are provided as a zip archive of all the generated results by *TSA-CRAFT*. This archive contains all previously generated results (html result page with relative paths and related graph overlays) and therefore can be used in a local environment.

We strongly suggest to download the results after the analyses as old queries results are cleaned from the server on a weekly basis.
